# Rigidity Percolation Dictates Rheological Hysteresis Regime in Polypropylene during Crystallization and Melting

**DOI:** 10.1021/acs.macromol.5c02223

**Published:** 2025-11-20

**Authors:** Paul Roberts, Chad R. Snyder, Anthony P. Kotula

**Affiliations:** Materials Science and Engineering Division, 10833 NIST, Gaithersburg, Maryland 20899, United States

## Abstract

Understanding structure–property relationships during polymer crystallization and melting has been limited by challenges in the simultaneous measurement of crystallinity and rheological properties. Consequently, rheological models overlook the fundamental asymmetry between crystallization and melting processes. Here, we use simultaneous rheology and Raman spectroscopy to directly measure rheological behavior as a function of crystallinity. We find that polypropylene’s rheological behavior can differ significantly between crystallization and melting at identical crystallinity values depending on thermal pathway. Using a generalized effective medium (GEM) model, we show that the onset of hysteresis aligns with the calculated percolation threshold. We quantify hysteresis through a normalized hysteresis parameter Δ*G̃* and show that the maximum value of Δ*G̃* occurs at the percolation threshold calculated by the GEM model for systems that have achieved complete space filling. Finally, we identify two hysteresis regimes: one prior to percolation with limited hysteresis and one after percolation with large hysteresis values. Mechanically, these regimes reflect the structural differences between the semicrystalline components and pure melt state: the former represents a suspension of “softening spheres” while the latter constitutes a softening network.

## Graphical abstract

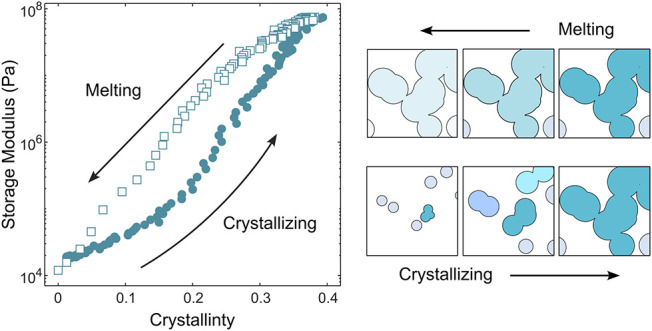

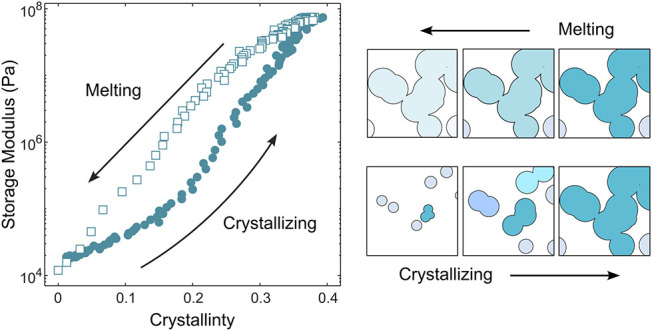

## Introduction

Semicrystalline polymers, particularly polyolefins, are ideal candidates for industrial processing and manufacturing; they flow readily when molten and can crystallize rapidly to form strong, durable materials. However, semicrystalline polymers are exceptionally process history dependent. Processing conditions such as degree of undercooling, cooling rate, and strain rate are all known to dramatically change the crystallization behavior of semicrystalline polymers and impact mechanical properties like strength and flexibility.^1–5^ Strain rate can have a drastic effect on polymer crystallization, enhancing nucleation density and modifying crystal geometry.^6,7^ The rate at which the molten material solidifies is also important; the rate and degree of undercooling can dramatically affect the spherulite size and specific volume of the semicrystalline polymer.^8^ Similarly, the difference between the equilibrium melting temperature and the crystallization temperature, often referred to as degree of undercooling, has been shown to affect spherulite size, nucleation density, melting temperature and, for some polymers, the distribution of resulting crystal polymorphs.^9,10^

Processing conditions also affect structure at larger length scales, affecting the size, number density, and prevalence of different semicrystalline morphologies ranging from spherulites to shish-kebabs. Consequently, it is the microscale aggregate structures of these semicrystalline morphologies that are paramount to the overall mechanical properties of the material. For example, larger spherulites typically result in materials prone to fracturing along the spherulite boundaries when stretched, while materials with smaller spherulites have greater fracture toughness.^11^ Semicrystalline structure growth and the formation of aggregated semicrystalline structures also affects the rheological properties of the material *during* crystallization,^12^ with rheological evolution that is sensitive to distinct semicrystalline morphologies (e.g., spherulites).^13^ Hyphenated techniques designed to measure the crystallinity–rheology relationship during crystallization have been implemented previously. Small-angle light scattering and polarized optical imaging techniques have been combined with rheology to relate structure to viscoelastic properties. Small-angle light scattering can give information about average spherulite size and can be used to characterize spherulite growth rates,^14^ while turbidity measurements can provide a sensitive measure of precrystalline structure growth.^15,16^ Small and wide-angle X-ray scattering (SAXS and WAXS, respectively) have been used with optical shear cells that allow for measurements of semicrystalline structure and orientation in a prescribed shear flow and temperature and are often referred to as “rheo-SAXS” and “rheo-WAXS”; however, these shear cells do not measure the torque required for a given rotation rate and do not measure viscoelastic properties. Small-angle scattering measurements have been successfully combined with rheological techniques to characterize structure–process–property relationships for a wide variety of complex fluids,^17^ but comparatively few of these techniques have been applied to the challenge of polymer melt crystallization. The multipass rheometer (MPR) has been successfully coupled with synchrotron-based SAXS/WAXS measurements to provide structural insight at process relevant conditions.^18,19^ The MPR is more similar to a capillary rheometer than a rotational rheometer, however it can perform oscillatory experiments through a dual piston design, forcing the polymer through a slit at specified frequencies.^19^ More typical rheology measurements, like those on a rotational rheometer, have been coupled with concurrent SAXS to observe changes in lamellar-scale structure and orientation along with viscoelastic properties during the crystallization of isotactic polypropylene, however these measurements require extensive customization.^20,21^ While not used for polymer crystallization, recently SAXS has been implemented along the flow-vorticity plane in a parallel plate rheometer to study particle clustering and anisotropy in polymer nanocomposites.^22^ While informative, these experimental set-ups can often require customized instrumentation at synchrotron user facilities. In contrast, Raman spectroscopy enables quantification of crystallinity while requiring minimal adaptation of the benchtop rheometer, making simultaneous rheological and Raman measurements (rheo-Raman spectroscopy) a readily accessible technique for quantifying crystallinity–rheology relationships.^23^

Given the inextricable nature of rheological properties on processing conditions and processing conditions on rheological properties, it is of great interest to relate moduli to relative crystallinity and several models have attempted to do so.^24–26^ Recently, a generalized effective medium (GEM) model has been shown to capture the frequency-dependent crystallinity–rheological behavior of poly-ε-caprolactone (PCL) during crystallization.^27^ A noted feature of the GEM model is that it accounts for the crystallinity at which rigidity percolation occurs (where the sample exhibits a critical gel response^28^) and describes viscoelastic properties across the percolation transition. This model can describe experimental results for polymer crystallization but assumes that the crystalline domains act as solids with constant moduli during the crystallization process.

Given the percolation-type process that occurs during crystallization, a critical question is whether these same concepts can be applied to melting. Melting is a common step in the thermal welding of semicrystalline polymers, which can occur multiple times during the formation of a part via advanced manufacturing techniques like material extrusion additive manufacturing,^29^ laser transmission welding^30^ and overmolding.^31,32^ The weld strength increases as polymer chains diffuse across the weld interface, but chain mobility is hindered by crystallinity. In models for polymer welding, polymer chain dynamics are assumed to depend on temperature. Material extrusion processes provide additional complexity as the polymer can periodically be reheated above its melting temperature. Although models can accommodate nonmonotonic temperature profiles,^33^ these models do not account for properties that depend on whether the crystallinity is increasing or decreasing at the time.^34^

Since polymer chain dynamics and rheology are closely related to morphology across multiple length scales, we can examine the well-known structural changes that govern crystallization and melting to determine whether the rheology during melting should be different from crystallization. Isothermal polymer crystallization from the melt state proceeds from the nucleation and growth of spherulites. Within this Avrami process, both the growth rate and the average thickness of the crystalline lamellae comprising each spherulite are constant and set by the degree of undercooling.^35^ The spherulites impinge to form larger semicrystalline superstructures^12^ that eventually span the sample to form a viscoelastic solid structure. The melting of semicrystalline polymers is often performed during a temperature ramp, and this melting process is well-described by a Gibbs–Thomson effect, where thinner lamellae melt at lower temperatures. However, there is a distribution of lamellae sizes throughout a given spherulite, implying that melting thinner lamellae will not necessarily break down the larger scale morphology. Indeed, small-angle light scattering measurements indicate that spherulites remain at a constant size during the melting process.^14,36^ Thus, the crystallization and melting processes are governed by structural changes over dramatically different length scales: crystallization proceeds via the growth of spherulites of size order 10 μm, while melting occurs via the destruction of thinner lamellae order 1 to 10 nm. This motivates a more detailed quantification of the relationship between crystallinity and rheological properties during crystallization and melting to improve our understanding of chain dynamics and relaxation time scales for these processes.

Here we show that the rheological properties of polypropylene depend on crystallinity *in addition* to whether the sample is crystallizing or melting. We refer to this phenomenon—a material’s ability to have different moduli at the same degree of crystallinity depending on thermal directionality (crystallizing or melting)—as “crystallinity–rheological hysteresis”. We use a rotational rheometer coupled with Raman spectroscopy to simultaneously measure rheological properties and crystallinity.^23^ Leveraging this capability, we show that a significant hysteresis occurs in the rheology of polypropylene as a function of crystallinity during crystallization and melting, and that the magnitude of this hysteresis depends on the degree of crystallinity attained prior to melting. This hysteresis is more apparent when melting samples that have crystallized in excess of the critical percolation fraction as parametrized from the GEM model. We develop a normalized hysteresis parameter to quantify the magnitude of this hysteresis and find that the storage modulus during melting can be approximately an order of magnitude larger than the storage modulus during crystallization for equal degrees of crystallinity. We discuss this hysteresis in the context of the different structural changes governing the rheology of the crystallization and melting processes.

## Experimental Section

### Materials

We use nucleating and clarifying agent free polypropylene (PP), as specified by the manufacturer. The number-average molar mass, mass average molar mass, and z-average molar mass are *M*_n_ = 20 kg/mol, *M*_w_ = 171 kg/mol, and *M*_z_ = 420 kg/mol, respectively. The resulting dispersity (*M*_w_/*M*_n_) is *D̵* = 8.4. The polymer is used as-received.

### Rheo-Raman Spectroscopy

We perform our experiments on a previously reported custom-built rheo-Raman instrument.^23^ A few noted modifications: we used an Anton-Paar MCR502 rotational rheometer coupled via fiber optic probe with a Thermo Fisher DXR Raman microscope equipped with a 780 nm laser source (see Disclaimer^37^). Each Raman spectrum is an average of 4 captured spectra with each spectrum being captured over a period of 5 s, resulting in a sampling rate of approximately 3 Raman spectra per minute.

For shear modulus measurements, we used an 8 mm diameter parallel plate measurement geometry to apply oscillatory shear. We used a 0.4% strain with a frequency of 1 Hz in the molten state, switching to a 0.01% strain in the semicrystalline state. All experiments begin with 5 min at 200 °C to erase thermal history. Then temperature is decreased at a rate of 5 °C/min until a temperature of 160 °C is reached. From 160 °C, temperature is further reduced at a rate of 1 °C/min until a temperature of 140 °C is reached. Temperature is kept constant at 140 °C until the desired shear modulus corresponding to an approximate degree of crystallinity is achieved. The temperature is then increased at a rate of 1 °C/min to melt the crystallized polymer. The heating rate (melting) was chosen to ensure accurate measured sample temperatures.

### Raman Spectra Deconvolution for Crystallinity

To determine the crystallinity of PP we perform peak deconvolution on measured Raman spectra. Representative Raman spectra of PP in the melt and semicrystalline states are shown in Figure 1a,b, respectively.

**Figure 1:**
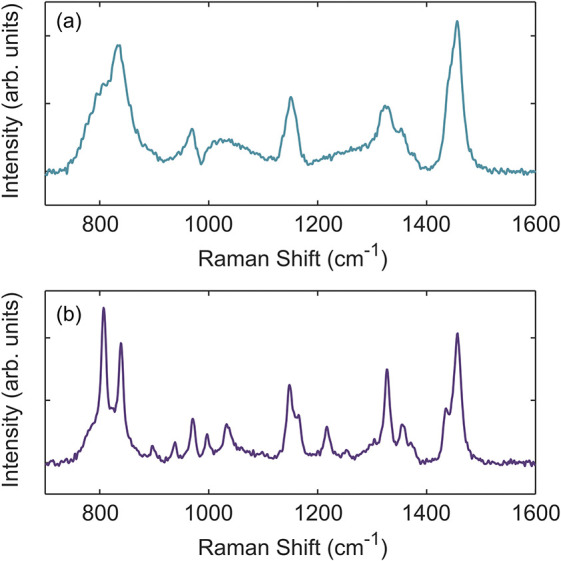
Raman spectra of PP at (a) 200 °C (melt) and at (b) 140 °C after ≈3900 s (semicrystalline). Spectra shown above have had a linear baseline fit over 700 to 1600 cm^–1^ subtracted from the raw captured spectra. Deconvolution of the 800 to 900 cm^–1^ region is shown in Figure 2.

We focus on the 800 to 900 cm^–1^ portion of the Raman spectrum. In the melt phase there is a predominant peak around 830 cm^–1^ corresponding to PP chains that are in a nonhelical conformation.^38^ Initial efforts to calculate crystallinity using the recommended peak fitting procedure in ref 38 were unsuccessful when compared with DSC measurements in the experimental temperature range, and we therefore modified the fitting procedure to obtain a satisfactory fit of the Raman spectra in the temperature range of 140 to 200 °C. To fit the melt component, we use the captured Raman spectra at 200 °C. We use two peaks to fit a general “melt spectrum” to this data, a Gaussian peak at 801 cm^–1^ and a Lorentzian peak at 835 cm^–1^. We then linearly scale each component of the spectrum when determining the crystallinity. However, we do not change peak position or width. In the semicrystalline state there are two strong peaks at 808 cm^–1^ and 841 cm^–1^, where the 808 cm^–1^ peak corresponds to helical chains and the 841 cm^–1^ peak corresponds to helical chains where the trans–gauche conformation is disrupted. Accordingly, to determine crystallinity we fit two Lorentzian peaks, one centered at 808 cm^–1^ and one centered at 841 cm^–1^. We use a least-squares fitting algorithm to fit all peaks in the measured Raman spectrum. Figure 2a,b shows deconvoluted melt and semicrystalline spectra.

**Figure 2:**
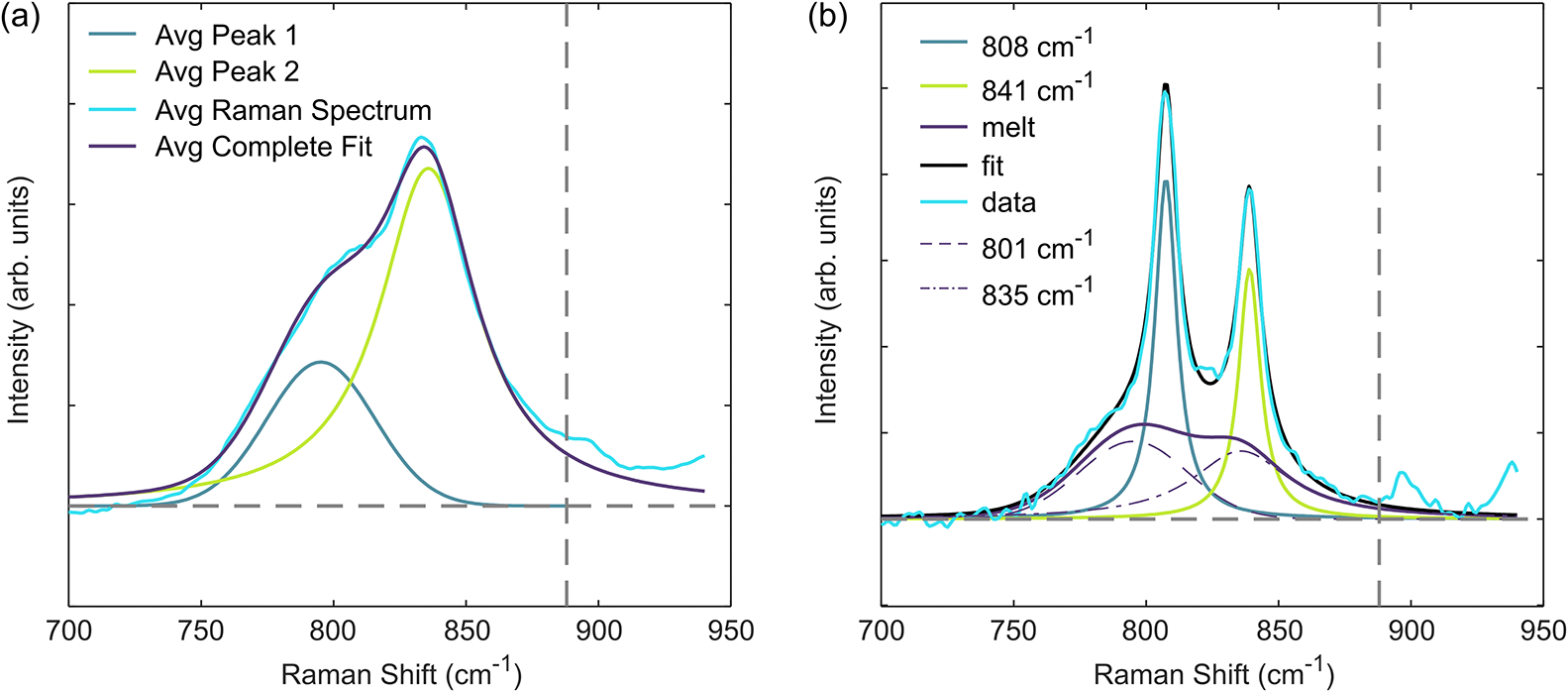
Peak deconvolution of PP to assess crystallinity. (a) Melt at 200 °C and (b) semicrystalline at 140 °C after 3900 s. Purple dashed and dot-dashed lines represent the two melt peaks that compose the melt component, purple solid line. Vertical dashed line indicates cut off where peaks are fit up to 700 to 888 cm^–1^. The area of each peak (as fit) is calculated over the entire range of the fit plotted 700 to 940 cm^–1^. The spectrum shown in (b) results in crystallinity *α* = 0.38 after converting between Raman crystallinity and mass fraction crystallinity.

To determine crystallinity, we use the area of 808 cm^–1^ compared to the combined 808 cm^–1^, 841 cm^–1^, and melt peaks, eq 1. Where the intensity of the melt component is *I*_melt_ = *I*_801_ + *I*_835_.

1 $$\alpha_{Raman}=\frac{I_{808}}{I_{808}+I_{melt}+I_{841}}$$

### Differential Scanning Calorimetry (DSC)

To convert between Raman crystallinity *α*_Raman_ and mass fraction crystallinity *α* we use a linear relationship, *α* = *βα*_Raman_. We use differential scanning calorimetry (DSC) to measure mass fraction crystallinity. To construct our curve, we anneal PP at 4 different temperatures after crystallizing at 140 °C for 90 min. The annealing temperatures (*T*_ann_) we use are 160 °C, 167 °C, 168.5 °C, and 170 °C, and we anneal until the Raman-measured crystallinity plateaus, or for 4 h for the DSC experiments. The exact temperature protocol followed for the DSC measurements (chosen to replicate the Raman measurements) was (1) equilibrate at 200 °C and isotherm for 5 min, (2) ramp −5 °C/min to 150 °C, (3) ramp −1 °C/min to 140 °C, (4) isotherm 90 min at 140 °C, (5) ramp 1 °C/min to *T*_ann_, (6) isotherm 240 min at *T*_ann_, (7) ramp −5 °C/min to *T*_ann_ – 5 °C, (8) isotherm 1 min at *T*_ann_ – 5 °C, and (9) ramp 10 °C/min to 200 °C. Steps 7 and 8 were added to enable capturing of the full melting endotherm. Figure S1 is a plot of step 9 for each of the annealing temperatures. The DSC measurements were performed on a TA Instruments DSC 2500 equipped with a refrigerated cooling system under a dry N_2_ purge.

By using different annealing temperatures, we are able to control the crystallinity prior to melting, are able to limit recrystallization during melting, and perform measurements in the temperature range relevant to our hysteresis experiments. The resulting crystallinity measurements are plotted in Figure S2 along with a corresponding linear least-squares fit. The linear fit yields *β* = 1.30 ± 0.02, the reported error is the standard error of the fit. We stress that this fit is valid in the temperature range of our experiments.

## Results and Discussion

The isothermal crystallization process can be described as a rigidity percolation process as shown in Figure 3. Crystallization begins with the formation of nuclei which subsequently grow outward, converting the melt into a semicrystalline solid. The mechanism of this phase transformation is characteristic of a nucleation and growth process and can be described by Avrami kinetics.^39–42^ Here, measurements of the viscoelastic moduli and crystallinity are measured simultaneously as a function of time during crystallization at 140 °C, then collated. We measure the crystallinity from the Raman spectra collected during the experiment.^37^ Storage and loss moduli show a dramatic increase with increasing relative crystallinity *ξ*, which is equal to the volume fraction crystallinity *ϕ* normalized by the crystallinity *ϕ*_∞_ at which spherulites have filled the sample domain. Although not directly measured here, the crystallization occurs in the temperature range expected to form the *alpha* polymorph.^43^ As the nucleated spherulites grow radially they eventually impinge to form a stress-supporting network.^35^ An appropriate model for such a process is the GEM model, which has been shown to capture polymer crystallization behavior well and can determine the critical gel point at which rigidity percolation occurs.^12,27,44^ The GEM model is an interpolation formula for binary mixtures using the property of interest for both individual components, interpolating between the pure phases for each component. A key requirement for implementing the GEM model is determining the degree of space filling. Here, we determine degree of space filling through measuring polymer crystallinity. To translate crystallinity to space filling we first use the measured crystal mass fraction *α* to calculate the crystal volume fraction of the semicrystalline phase *ϕ* (eq 2), where *v*_m_ is the specific volume of the melt phase, *v*_c_ is the specific volume of the semicrystalline phase. We use *v*_m_ of 1.3210 cm^3^/g as it is the specific volume of the melt phase before crystallization (specific volume of PP at 180.7 °C) and *v*_c_ of 1.1865 cm^3^/g, the specific volume of PP at 140 °C (our isothermal crystallization temperature).^45^

2 $$\phi =\frac{\alpha }{\alpha +\left(\frac{v_{m}}{v_{c}}\right)\left(1-\alpha \right)}$$

Then we rescale volume fraction to vary between 0 and 1 by eq 3, where *ξ* is relative crystallinity and *ϕ*_∞_ is the crystallinity corresponding to *G*_∞_^*^.

3 $$\xi =\frac{\phi }{\phi_{\infty }}$$

We can then use the GEM model (eq 4) to calculate the critical relative crystallinity *ξ*_c_, the relative crystallinity at which percolation occurs. Equation 5 defines *A*.

$$\begin{array}{c} \left(1-\xi \right)\frac{{\left(G_{0}^{*}\left(\omega \right)\right)}^{1/s}-{\left(G^{*}\left(\omega \right)\right)}^{1/s}}{{\left(G_{0}^{*}\left(\omega \right)\right)}^{1/s}+A{\left(G^{*}\left(\omega \right)\right)}^{1/s}}\\ +\xi \frac{{\left(G_{\infty }^{*}\left(\omega \right)\right)}^{1/t}-{\left(G^{*}\left(\omega \right)\right)}^{1/t}}{{\left(G_{\infty }^{*}\left(\omega \right)\right)}^{1/t}+A{\left(G^{*}\left(\omega \right)\right)}^{1/t}}=0 \end{array}$$ 4

5 $$A=\frac{1-\xi_{c}}{\xi_{c}}$$

Here, *G*_0_^*^ is the complex shear modulus at zero crystallinity, *G*_∞_^*^ is the complex shear modulus at complete space filling, G* is the complex modulus at a degree of space filling *ξ*, and the relative crystallinity at which percolation occurs *ξ*_c_. Note that each complex modulus consists of a storage modulus *G*′ and a loss modulus *G*″ since G* = *G*′ + i*G*″; the GEM model fits both *G*′ and *G*″ simultaneously.

**Figure 3:**
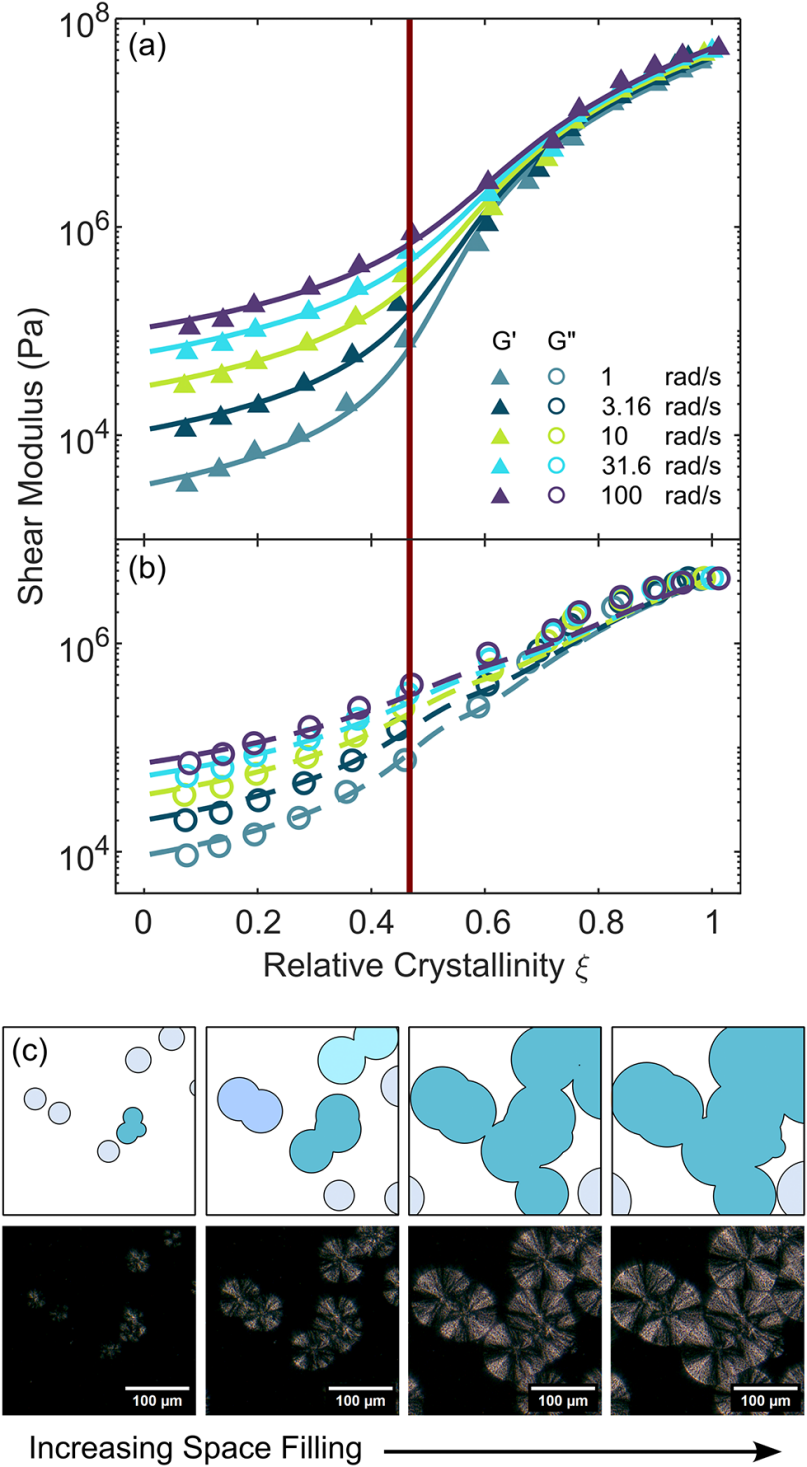
GEM for critical percolation and corresponding *ξ*. (a) *G*′ and (b) *G*″ from a multifrequency GEM fit for PP isothermal crystallization at 140 °C. Each color is a different frequency logarithmically spaced between 1 and 100 rad/s. (c) Schematics and images display the geometric meaning for different *ξ* relative to *ξ*_c_.

Examining the model at idealized limits for *G*_0_^*^ and *G*_∞_^*^ reveals additional insight into the material system, as discussed in detail by Kotula and Migler in ref 44. In the limit where *G*_∞_^*^ goes to infinity, eq 4 simplifies to $G^{*}=G_{0}^{*}{\left(1-\xi /\xi_{c}\right)}^{-s}$ , which resembles a Krieger–Dougherty relationship for a material consisting of hard particles dispersed in a viscoelastic matrix. The scaling exponent *s* has been shown to characterize the concentration dependent rheological behavior of colloidal suspensions. For a suspension of rigid spheres *s* should be approximately 2, as seen in colloidal systems. From ref 44, the exponent *s* can be related back to the critical percolation fraction and the (dimensionless) intrinsic shear modulus [*G*] via the relationship *s* = [*G*]*ξ*_c_. The intrinsic shear modulus is related to the shape, shear modulus, and deformability of the dispersed phase, which in this case are the spherulites growing from the melt.^46^ In this simplified model, the modulus G* of the composite approaches infinity as the particle loading increases to the critical percolation fraction. If instead we set *G*_0_^*^ = 0 in the GEM model, we recover a phenomenological equation describing the viscoelastic response of a solid matrix with voids: $G^{*}=G_{\infty }^{*}{\left(\left(\xi -\xi_{c}\right)/\left(1-\xi_{c}\right)\right)}^{t}$ . Here, the modulus of this porous solid approaches zero as the volume fraction of the solid matrix decreases to *ξ*_c_. Analogous to the value of *s* in the Krieger–Dougherty relationship, the value of *t* can provide information on the postpercolation structure. Here, for the case of a solid matrix with voids, increasing values of *t* generally indicate increased interconnectivity of the melt domain after percolation.^44^ The GEM model provides a phenomenological model that describes the viscoelastic response between these two limits. We also note that the GEM model can reproduce the power law relaxation dynamics expected for critical gels where *ξ* = *ξ*_c_, which was observed in the crystallization of polypropylenes in the work of Pogodina and Winter^28^ and is further discussed in refs 27 and 47 .

The GEM model fit to isothermal crystallization data at 140 °C is shown in Figure 3a,b. The fit results in *ξ*_c_ = 0.49 ± 0.14, *s* = 1.24 ± 0.19, and *t* = 3.35 ± 1.26 with reported uncertainties based on the curve fit. Using the results from the GEM model fit and the relationship *s* = [*G*]*ξ*_c_, we find [*G*] = 2.5, which is the expected value for rigid spherical particles.^46^ The *ξ*_c_ returned by our fit is 0.49, which corresponds to a crystalline mass fraction of *α*_c_ = 0.18. This is larger than the previously reported *ξ*_c_ for PP crystallizing isothermally based on simultaneous rheology and optical microscopy measurements, which was reported to be *ξ*_c_ = 0.37.^12^ However, there are differences in the molar mass and dispersity between the PP in ref 13 (*M*_w_ = 448 kg/mol and *D̵* = 7.2) and that used here (*M*_w_ = 171 kg/mol and *D̵* = 8.4), and the effect of these parameters on *ξ*_c_ is unknown. Additionally, PP has been shown to have a higher mass fraction crystallinity in the center of its spherulites as compared to the spherulite boundary.^48^ This, in turn, means that a given spherulite will occupy a different volume than expected from a crystallinity to volume calculation due to the difference in density, causing *ξ*_c_ measured by volume fraction crystallinity to differ from *ξ*_c_ via direct spherulite imaging. Furthermore, the impact of modulus gradients on the volume fraction separating geometric and rigid percolation is unknown, geometric percolation being when a space spanning network is formed and rigidity percolation being a *stress-supporting* space spanning network. Geometric percolation necessarily occurs simultaneously or prior to rigidity percolation.

Figure 3c shows schematics and polarized optical images of the Avrami process for the crystallizing polymer measured on a separate temperature stage and optical microscope. The optical images provide information on geometric percolation, which is known to occur before rigidity percolation (*ξ* = *ξ*_c_).^49^ Since spherulites are the dominant rheology modifier for crystallizing polymers^12^ these images provide an approximation of the rigidity percolation process. Prior to percolation spherulites remain distinct, and as *ξ* approaches *ξ*_c_ some spherulites impinge on each other creating multispherulite structures of finite size. At *ξ*_c_ a stress-supporting network is formed. At *ξ* larger than *ξ*_c_ spherulites or smaller spherulite clusters can continue to join the network, but largely the network continues to crystallize outward where there is space available.

To quantify the modulus–crystallinity relationship during melting, we first crystallize PP isothermally at 140 °C to various partially crystalline states and subsequently melt the samples using a constant temperature ramp (Figure 4). We use *G*′ as a means of specifying the start of the melting ramp. Figure 4a shows the prescribed temperature profile as a function of time with simultaneous rheological and crystallinity measurements (Figure 4b,c, respectively). Shear moduli are measured using a single oscillation frequency of 6.28 rad/s, which allows for a greater number of modulus measurements to correlate with crystallinity during crystallization and melting.

**Figure 4:**
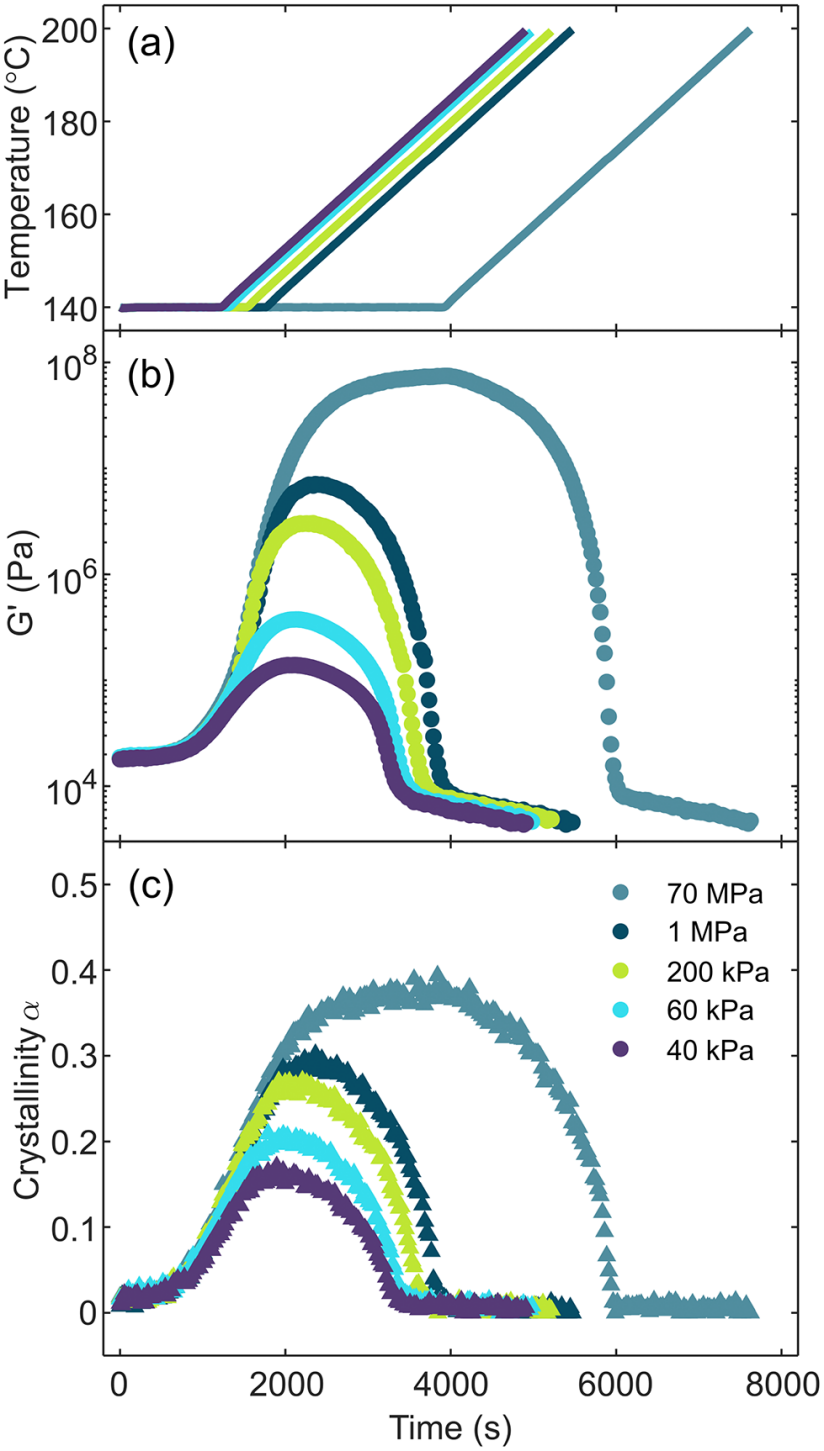
Simultaneous shear modulus and crystallinity measurements during crystallization and melting of PP. (a) Measured temperature, (b) shear storage modulus, and (c) crystallinity as a function of time. Experiments are labeled by the *G*′ threshold at which isothermal crystallization is stopped and the melting ramp begins, where 70 MPa is a case of complete space filling prior to melting. For all cases where melting occurs before complete space filling is achieved, the increase in *G*′ and crystallinity immediately after the melting ramp beings is due to PP’s ability to continue to crystallize at the initial temperatures in the melting ramp. *G*′ is measured at 6.28 rad/s with a strain amplitude of 0.004 in the melt state and 10^–4^ in the semicrystalline state (see Experimental Section for more detail).

Holding the sample at the crystallization temperature, *T*_c_, for extended durations results in a rapid increase of both crystallinity and viscoelastic moduli until a slower growth process attributed to secondary crystallization occurs after approximately 2000 s.^48^ During the temperature ramp from this crystallization condition, both modulus and crystallinity decrease sharply with increasing temperature until approximately 170 °C when the sample melts. Changing the duration that the sample remains in this secondary crystallization process does not affect this trend (see Supporting Information Figures S3 and S4). There is a clear correlation between the crystallinity and rheological properties of the sample during both crystallization and melting.

To investigate whether the rheology of the melting samples depends on the extent of crystallinity, we interrupt the isothermal crystallization of PP both above and below the percolation threshold. Experimentally, this interruption simply means that we prematurely end crystallization by beginning to melt the sample before it has achieved complete space filling (Figure 4a). Figure 4 shows that by modulating the amount of time we spend at *T*_c_ we are able to control the resulting maximum degree of crystallinity achieved prior to melting. We note that Figure 4 shows that for all partial crystallization experiments crystallinity continues to increase immediately after we begin to increase the temperature. This is due to semicrystalline polymers’ ability to crystallize across a range of temperatures, both above and below the *T*_c_ chosen here. We also note that even though we achieve a large range of maximum crystallinities, 0.15 to 0.4, all samples appear to melt fully (reach zero crystallinity) at temperatures near 170 °C, as expected for the presumed *alpha* polymorph spherulites formed during the crystallization process.^43^

We plot the shear storage modulus as a function of crystallinity for different extents of crystallization in Figure 5a (three conditions are shown for clarity, all five data sets are plotted along with the individual hysteresis traces in the Supporting Information Figures S5–S10). Most noticeably, the crystallization behavior for every sample, regardless of maximum crystallinity achieved, follows the same crystallinity–modulus curve. The melting behavior, however, exhibits strong path dependency. Here we discuss the percolation threshold in terms of crystal mass fraction, *α*, since the condition where complete space filling occurs is unknown for the partially crystallized samples, nor can the same assumptions regarding space filling during crystallization be made during melting. We see that below *α*_c_ the crystallinity–modulus relationship during melting is largely the same as during crystallization, the curves fall on top of each other. However, the more *α* progresses beyond *α*_c_, the more crystallization–melting hysteresis is present, crystallization–melting hysteresis being the difference in modulus between crystallization and melting at the same *α*. We find the loss tangent to display crystallization–melting hysteresis as well, with the onset of hysteresis also appearing to operate about *α*_c_ (Figure S11). Further, we find that hysteresis is similar at slower melting rates (Figure S12), indicating this phenomenon is not due to thermal lag effects. The crystallinity–modulus hysteresis becomes more apparent when plotting the normalized difference between the crystallizing (*G*_c_^′^) and melting (*G*_m_^′^) shear moduli for a given degree of crystallinity (Figure 5b). We define this normalized hysteresis as

6 $$\Delta \mathrm{G̃}\left(\alpha \right)=\frac{G_{m}^{\prime }\left(\alpha \right)-G_{c}^{\prime }\left(\alpha \right)}{G_{c}^{\prime }\left(\alpha \right)}$$

**Figure 5:**
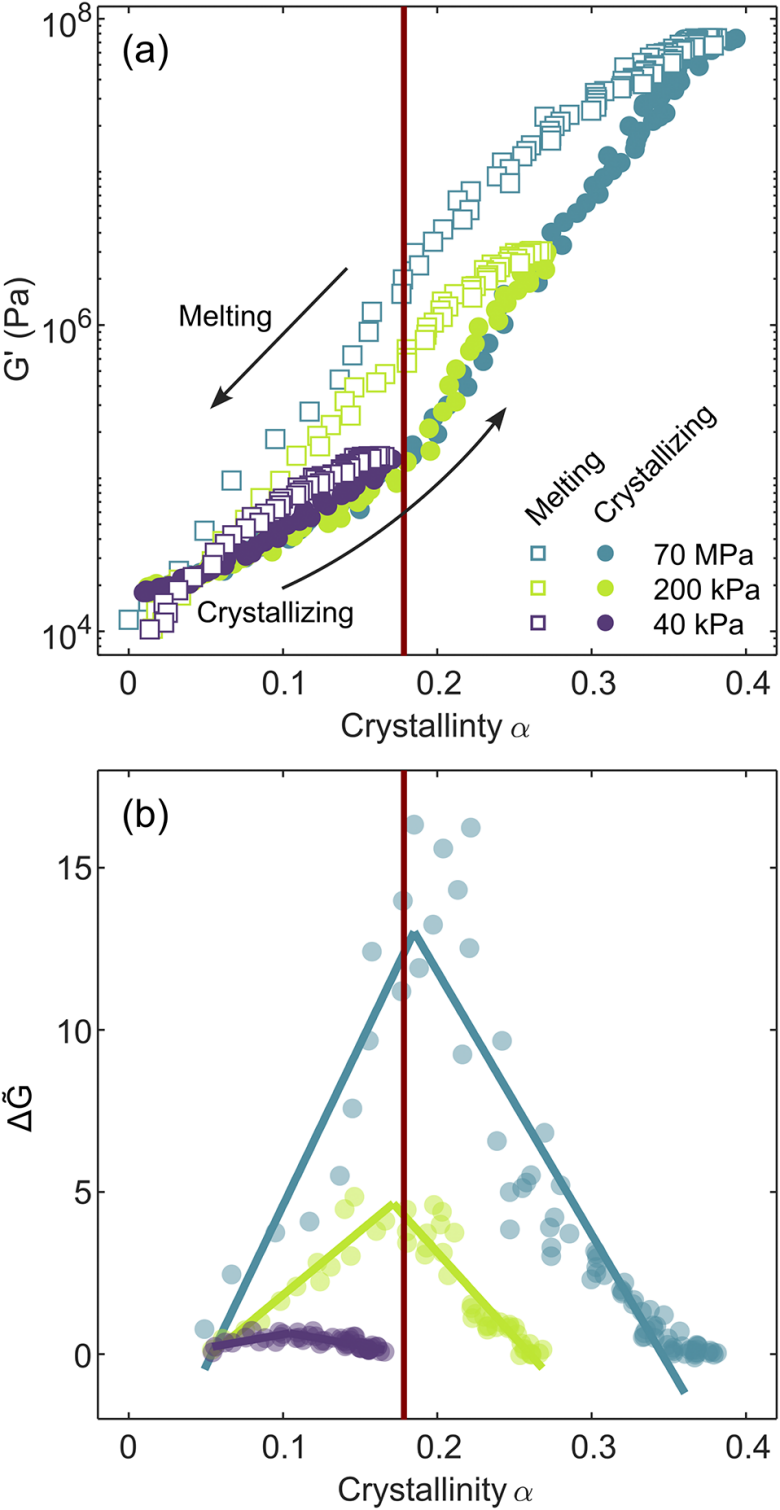
Rheology–crystallinity hysteresis of PP. (a) Shear storage modulus, *G*′, as a function of crystal fraction during crystallization (closed circles) and melting (open squares) with a variable maximum crystallinity. Three conditions are shown for clarity (b) Normalized hysteresis, the difference between *G*′ during melting and crystallization at a given crystallinity normalized by *G*′ during crystallization (Δ*G̃* = *G*_m_^′^/*G*_c_^′^ – 1), as a function of crystallinity. Negative values of Δ*G̃* are not shown. Solid lines are the fit from two component segmented linear regression. Critical crystallinity, *α*_c_, indicating the onset of percolation is shown as a red vertical line at *α*_c_ = 0.18. Experiments are labeled by the *G*′ threshold at which isothermal crystallization is stopped and the melting ramp begins, where 70 MPa is a case of complete space filling prior to melting. Rheology–crystallinity hysteresis loops for various heating rates are provided in the Supporting Information, Figure S12.

We see that as the maximum crystallinity reached in each experiment increases, so does the maximal value of Δ*G̃*, with a large change in maximal Δ*G̃* when the maximum crystallinity for an experiment surpasses *α*_c._ For the case of complete space filling we can see that Δ*G̃* displays a maximum at *α*_c._ This presents an alternative methodology to determine *α*_c_ (and *ξ*_c_) without the need for fitting a model. Furthermore, the substantial increase in hysteresis about *α*_c_ indicates that percolation plays a significant role in the crystallinity–rheology hysteresis between melting and crystallization of semicrystalline polymers. Since *G*_c_^′^ is measured isothermally and *G*_m_^′^ is measured during a temperature ramp, it is important to estimate the role of thermorheological effects on the melt rheology over the experimental temperature range. This is most easily observed at crystallinity values near 0 in Figure 5a, where the storage modulus during melting (near 170 °C) is lower than that during crystallization (at 140 °C) by approximately a factor of 2. Thus, the temperature sensitivity of the storage modulus in the melt state most likely reduces the magnitude of the hysteresis, but this contribution is generally smaller than the effect of the crystal-to-melt transition occurring during heating.

Melting is dictated by lamellar thickness. Thinner lamellae melt at lower temperatures while thicker lamella melt at higher temperatures; this is known as the Gibbs–Thomson effect.^10^ In a given spherulite there are a distribution of lamellar thicknesses, and not all lamella of a given thickness are at a specific radial coordinate nor are isothermally formed spherulites comprised of equally thick lamella. This melting behavior implies that the “macro” scale structure of the spherulitic network is maintained during melting, however the nanoscale spherulite interior is undergoing changes (melting of thinner lamella). We find that the observed hysteresis is not strongly affected by additional crystallization beyond complete space filling (Figures S3 and S4). In the context of mechanical properties, this means during melting we have a continually softening semicrystalline system. If we are below *ξ*_c_ (not percolated) during melting, we have a melt containing isolated softening spherulites. If we are above *ξ*_c_ (percolated) the system is a softening network.

The above description is in agreement with observations from polarized optical microscopy. Figure 6 shows polarized optical micrographs (bottom) during the melting of a partially crystallized polymer melt. In the first panel we can see impinged spherulites exhibiting a large degree of birefringence. As temperature is raised, the spherulites become less birefringent, directly indicating a decrease in crystallinity, with the thinnest lamella melting first (Figure 6b,c). The lamellae continue to melt from thinnest to thickest with increasing temperature until all lamellae are melted and no spherulites or birefringence is observed. However, the overall geometry of the system, whether it be individual spherulites or impinged spherulites, is maintained during this melting process. The corresponding schematics for the melting spherulites illustrate how thinner lamella melt before the larger lamella, however until *all* lamellae have melted a spherulitic structure endures.

**Figure 6:**
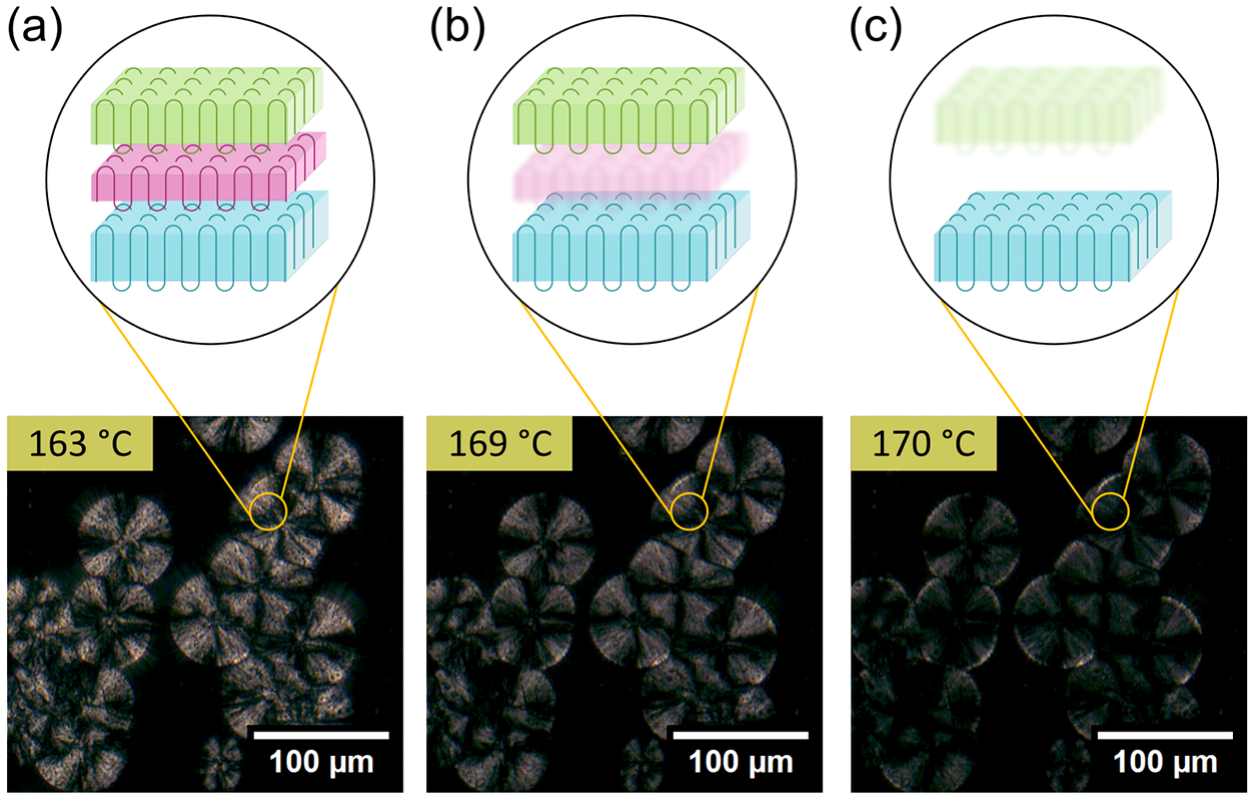
Spherulites maintain impingements during melting. Bottom row shows polarized optical micrographs of PP spherulites during a heating cycle where the black background is PP in the disordered melt state at temperatures (a) 163 °C, (b) 169 °C, and (c) 170 °C. Top row schematically illustrates how the lamellae in a spherulite melt during a heating cycle.

We note that little hysteresis is observed for lower crystal fractions; the viscoelastic properties of the partially crystalline material exhibit different sensitivities to the spherulite modulus depending on whether rigidity percolation has occurred in the sample. Below percolation, the viscoelastic modulus is affected more so by the volume fraction of spherulites than their stiffness, since the spherulite modulus is much greater than that of the matrix. Once the spherulites have formed a percolated network, however, the softening of those spherulites comprising the network has a dominating effect on the modulus of the melting sample.

Thus, far we have focused on a general spherulitic morphology. However, spherulites can have many multiple polymorphs, each with unique crystallization, melting, shape, and mechanical properties. Polypropylene, for example, is known to have *alpha*, *beta*, *gamma*, and smectic or mesomorphic polymorphs.^9,50^ These polymorphs are controlled by processing conditions and additives; for example, high shear and fast cooling rates favor mesomorphic phases.^51,52^

The relative growth rates of *alpha* and *beta* spherulites are also temperature-dependent. Above a *T*_c_ of 141 °C *alpha* spherulites grow faster while between 141 °C and approximately 100 °C *beta* spherulites grow faster.^50,53,54^ However, *beta* polymorphs nucleate more sparsely than *alpha*. These nucleation and growth rate differences could result in a bimodal distribution of spherulite sizes, which has been shown to increases the maximum packing fraction of spheres in a suspension^55,56^ and can increase the percolation threshold.^57^ However, in our polarized optical microscopy experiments we did not observe any spherulites with negative birefringence, a characteristic indicator of *beta* spherulites. Additionally, given our crystallization conditions and lack of *beta* nucleating agent, *beta* polymorphs are unlikely to form, leading us to believe that the presence of *beta* spherulites in our experiments is negligible. The resulting rheology–crystallinity melting behavior observed here is predominantly from *alpha* spherulites.

The structure, connectivity, and orientation of semicrystalline structures at spherulitic length scales will influence the critical percolation fraction and consequently Δ*G̃*. Compared to a homogeneous population of spheres, oriented prolate or oblate spheroidal structures can achieve a larger degree of space filling prior to percolation while randomly oriented spheroidal structures percolate at lower degrees of space filling.^46^ During heating, the structural integrity of these spheroids will decrease as lamellae within them melt, regardless of whether the spheroids were oriented during the crystallization process. As a result, we expect that percolation at lower degrees of space filling due to the nucleation and growth of randomly oriented anisotropic semicrystalline domains would lead toward lower values of Δ*G̃* near *ξ*_c_. In contrast, semicrystalline domains that are oriented by flow and therefore percolate at higher degrees of space filling would exhibit larger values of Δ*G̃* near *ξ*_c_.

The specific shape and magnitude of the hysteresis loop will likely vary depending on crystallization kinetics, molar mass distribution, polymer architecture, and thermal history. For example, different isothermal crystallization temperatures are likely to produce different magnitudes of hysteresis, with higher *T*_c_ achieving lower maximal crystallinities compared to lower *T*_c_. Additionally, *M*_w_ and *D̵* are expected to influence the hysteresis loop as well. Previously it has been shown that differences in *M*_w_ have little to no impact on *ξ*_c_.^27^ However, we expect that differences in *M*_w_ will affect the *G*′–crystallinity relationship when measured at the same temperature and frequency. Nevertheless, the underlying mechanism—the transition from dispersed spherulites to a stress-supporting network and back to a melt—remains broadly applicable. The established mechanisms of nucleation-and-growth crystallization and Gibbs–Thomson melting behavior have been shown in many semicrystalline polymers including polyethylene,^58^ polycaprolactone,^59^ and poly­(lactic acid).^60^ This further supports the generalizability of the crystallinity–modulus hysteresis described here beyond polypropylenes to other homopolymers crystallizing from an initial melt state. Characterizing the frequency-dependent response of this hysteresis is also of immediate interest. Although we have used a single frequency to demonstrate this hysteresis, advanced techniques such as optimally windowed chirps^61,62^ can be used to rapidly obtain viscoelastic moduli over a wide frequency range during both crystallization and melting.

Crystallinity–rheology hysteresis brings to light important considerations for additive processes like fused filament fabrication and overmolding, where polymer chain mobility governs interlayer diffusion and weld strength. As polymer chain mobility is inversely related to viscoelastic modulus,^63,64^ our findings imply that for the same degree of crystallinity a crystallizing polymer has a higher degree of chain mobility than a melting one. However, it is not obvious how this general implication translates to interlayer diffusion across weld layers for semicrystalline polymers, since the interdiffusion of chains across a weld interface will depend both on the volume fraction of mobile chains and the location of those polymer chains freed during the melting process. During crystallization there is a distinct melt-spherulite boundary, where polymers that have not crystallized nominally have the mobility of a polymer melt without any spherulites. However, during melting the thinner lamellae within partially melted spherulites will have their mobility influenced by the surrounding crystallites. Polymer mobility has been shown to impacted by interfaces^65^ and in crystallizing block copolymers mobility can be dependent on primary crystallite inclusion.^66^ Whether net mobility at a given crystallinity is well described by modulus between crystallization and melting, the impact of path dependent topologies across multiple length scales on interlayer diffusion, has yet to be fully explored and warrants further investigation.

## Conclusion

We have shown that there exists a substantial crystallinity–rheological hysteresis in a semicrystalline polymer during crystallization and melting. During melting it is possible to have a shear modulus more than an order of magnitude larger than the modulus of a crystallizing sample at the same crystallinity. By describing polymer crystallization through a percolation-type process model we are able to determine the onset of percolation, finding the onset to coincide with the advent of the observed crystallinity–rheological hysteresis. This finding agrees with both the suspension-based rheological modeling of the crystallization process and the current understanding of polymer crystallization and melting. During crystallization, polymer spherulites will grow radially, but when they melt, the smallest lamellae will melt first, and as temperature increases larger and larger lamellae will begin to melt leaving a “weaker” (less crystalline) spherulite that occupies its original volume. Impingements or a space spanning network are similarly maintained. Once percolation has occurred semicrystalline polymers will melt as a gradually softening network as opposed to a suspension. We believe this hysteresis occurs in most semicrystalline polymers, provided the polymer goes through two phases, one where discrete semicrystalline morphologies (e.g., spherulites) have yet to form a stress spanning network and one after aggregates of these morphologies have formed such a network. Specific polymer and morphological characteristics across multiple length scales are expected to impact the shape of the hysteresis loop and the magnitude of hysteresis, but ultimately the presence of hysteresis will be maintained. In addition to polymer processing and reprocessing, crystallinity–rheological hysteresis has broad applicability to strategies for mitigating residual stresses, engineering shape memory, polymer design, and additive manufacturing; once spherulites are impinged they must be completely melted otherwise the rheological properties of the percolated network will persist.

## Supplementary Material



## Notes

Additional crystallinity–rheology hysteresis curves and DSC–Raman crystallinity conversion details are provided in the Supporting Information (PDF)
