# Supplementary material for: Rigidity Percolation Dictates Rheological Hysteresis Regime in Polypropylene during Crystallization and Melting
Source: Macromolecules. 2025 Nov 20;58(23):12596–605. doi: 10.1021/acs.macromol.5c02223 (PMC12874635; doi:10.1021/acs.macromol.5c02223)
Supplement: Supplementary file 1 [file ma5c02223_si_001.pdf]

Supporting Information: Rigidity percolation dictates rheological hysteresis regime in polypropylene during crystallization and melting

Authors: Paul Roberts[1], Chad R. Snyder[1], Anthony Kotula[1]*

Affiliations:

[1]Materials Science and Engineering Division, NIST; Gaithersburg, Maryland 20899

*Corresponding author. Email: anthony.kotula@nist.gov

Copyright Disclaimer: Official contribution of the National Institute of Standards and Technology; not subject to copyright in the United States.

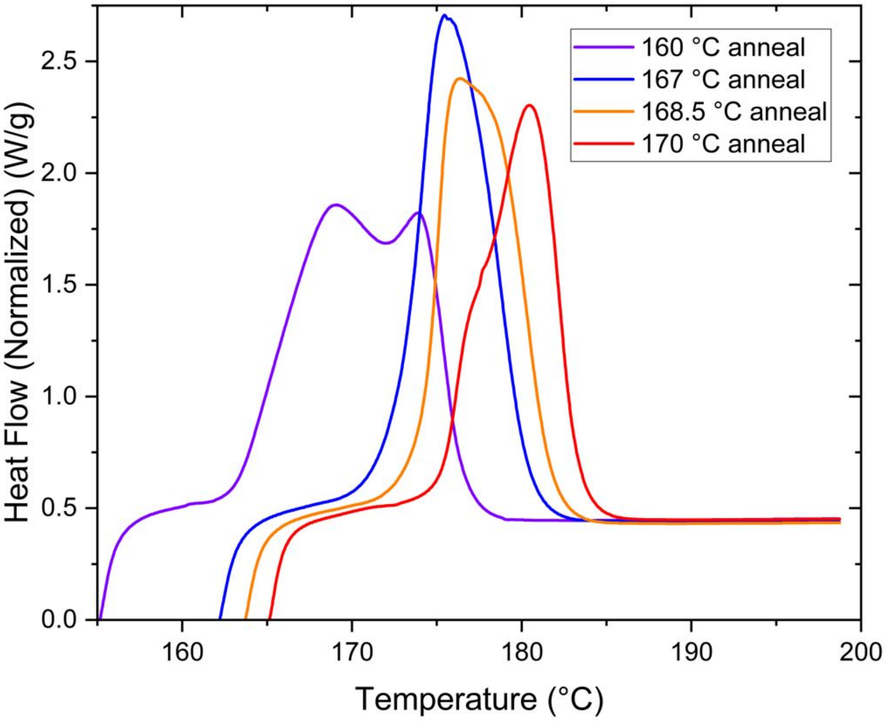

Figure S1. DSC scans for polypropylene samples annealed for 4 h at 160 °C, 167 °C, 168.5 °C, and 170 °C. (Note that the heating scans were performed after cooling to 5 °C below the annealing temperature to enable capturing of the full melting endotherm. See the text for additional details.) Endotherm direction is up.

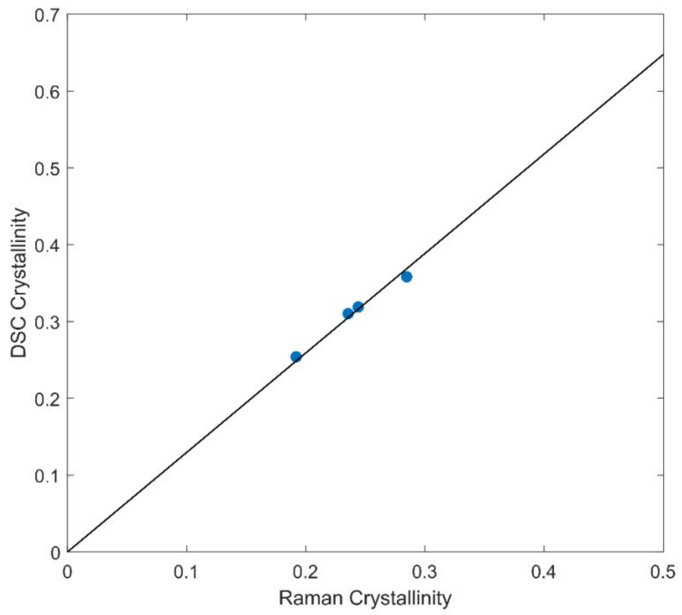

Figure S2. α_{Raman} as calculated from Equation 1 compared to DSC crystallinity for PP. The solid line displays the linear fit between α_{Raman} and DSC crystallinities that allows us to determine β as described in the Experimental Section.

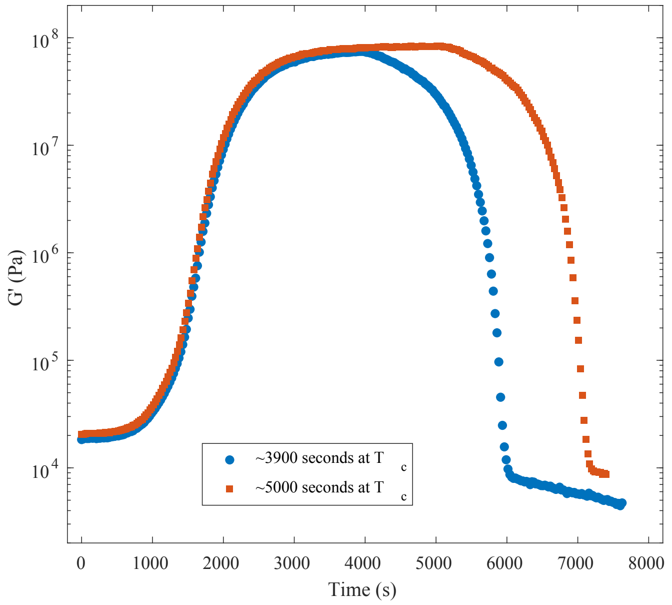

Figure S3. Two isothermal crystallization experiments at T_c of 140 °C followed by heating at 1 °C/min. The experiment represented by the blue circles crystallizes at T_c for approximately 3,900 s while the experiment represented by the orange squares crystallize at T_c for approximately 5,000 s. Longer times at T_c are expected to yield more secondary crystallites.

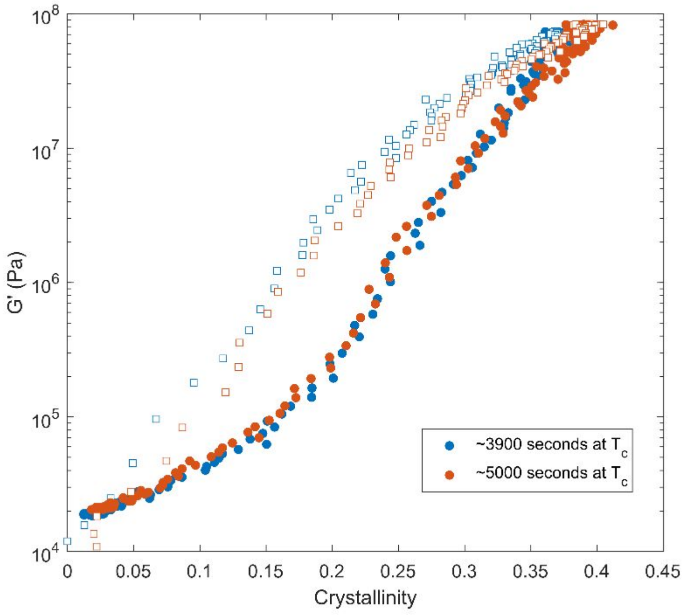

Figure S4. Rheology-crystallinity hysteresis plots for two different crystallization times. The plot shows the shear storage modulus G' as a function of crystallinity during crystallization (closed circles) and melting (open squares) for the two experiments shown in Fig. S3. The melting G' vs crystallinity data for both experiments is similar, indicating that secondary crystallization is not contributing significantly to the observed rheology-crystallinity hysteresis.

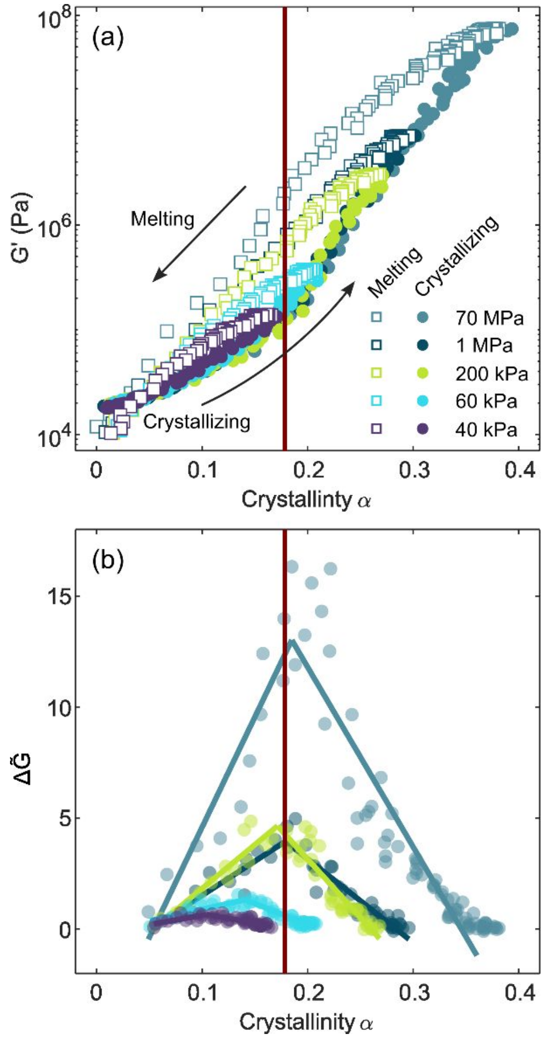

Figure S5. Rheology-crystallinity hysteresis for five experimental conditions. Critical crystallinity, α_c, indicating the onset of percolation is shown as a red vertical line at $\alpha_c = 0.18$.

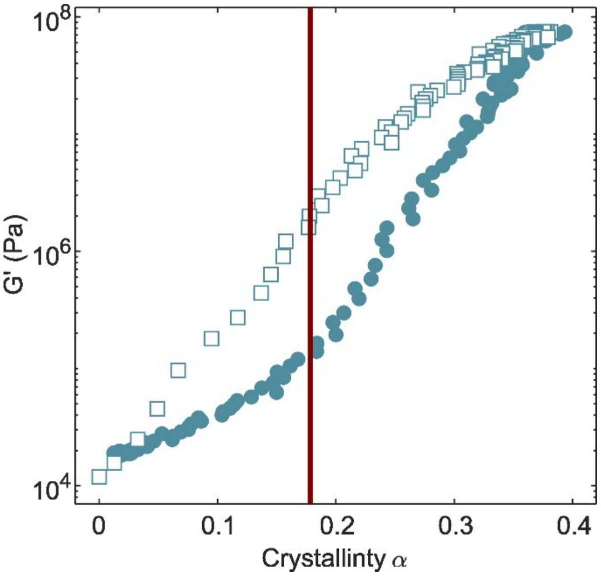

Figure S6. Rheology-crystallinity hysteresis for complete isothermal crystallization. Critical crystallinity, α_c, indicating the onset of percolation is shown as a red vertical line at $\alpha_c = 0.18$.

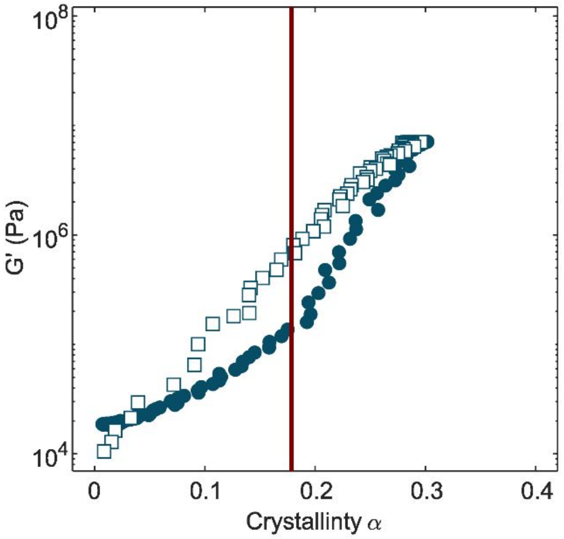

Figure S7. Rheology-crystallinity hysteresis where melting ramp begins when $G' > 1$ MPa at T_c. Critical crystallinity, α_c, indicating the onset of percolation is shown as a red vertical line at $\alpha_c = 0.18$.

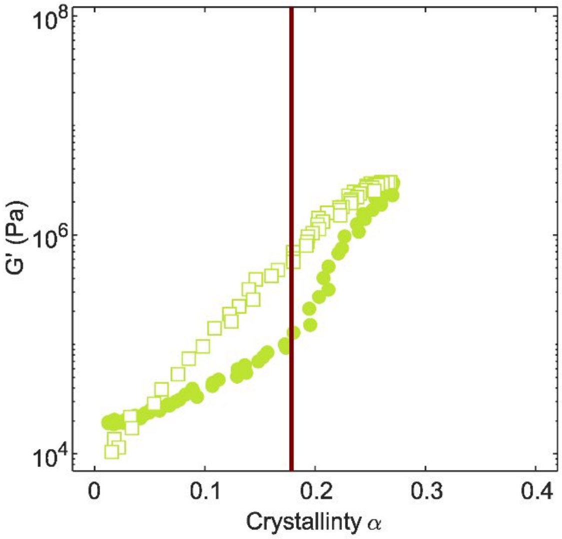

Figure S8. Rheology-crystallinity hysteresis where melting ramp begins when G' > 200 kPa at T_c. Critical crystallinity, α_c, indicating the onset of percolation is shown as a red vertical line at α_c = 0.18.

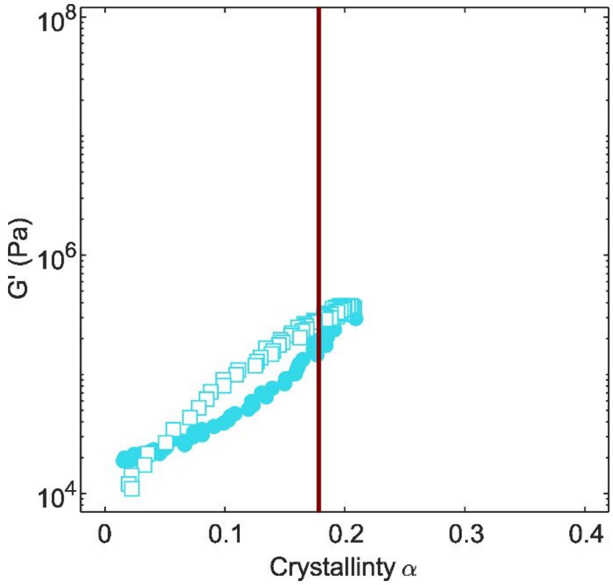

Figure S9. Rheology-crystallinity hysteresis where melting ramp begins when G' > 60 kPa at T_c. Critical crystallinity, α_c, indicating the onset of percolation is shown as a red vertical line at α_c = 0.18.

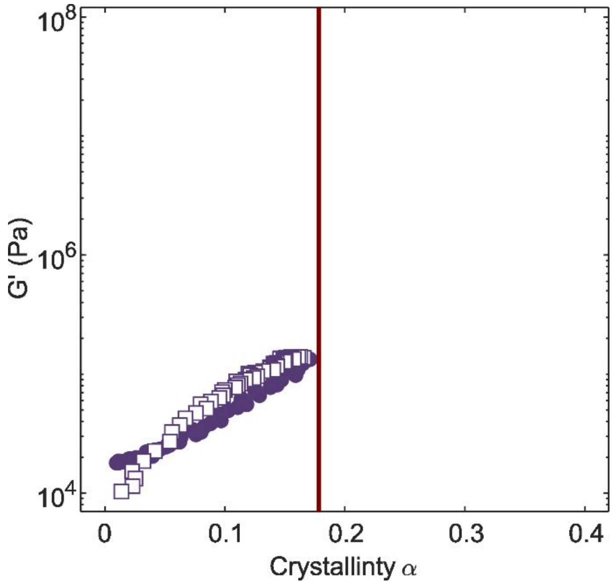

Figure S10. Rheology-crystallinity hysteresis where melting ramp begins when $G' > 40$ kPa at T_c. Critical crystallinity, α_c, indicating the onset of percolation is shown as a red vertical line at $\alpha_c = 0.18$.

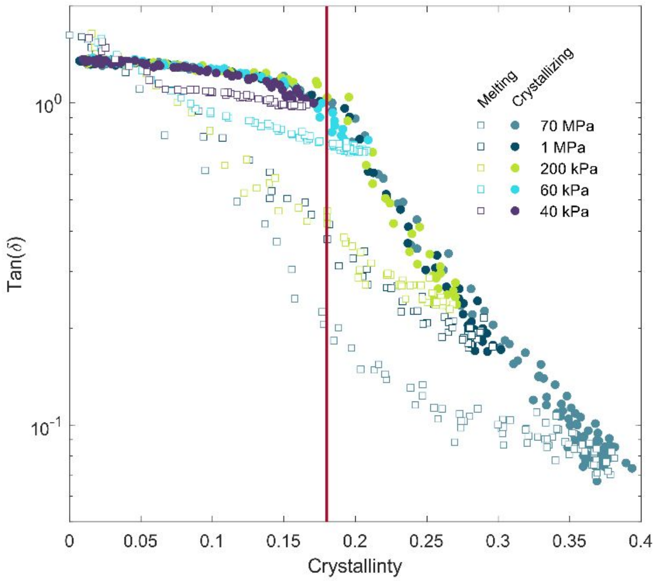

Figure S11. Loss tangent tan(δ) hysteresis plot. There exists hysteresis in both the storage and loss moduli. Similar to Fig. 5, the calculated percolation condition using the GEM model, $\alpha_c = 0.18$, delineates a change in slope of the crystallization curve, as well as a change in slope of the resulting melting curve. Critical crystallinity, α_c, indicating the onset of percolation is shown as a red vertical line at $\alpha_c = 0.18$.

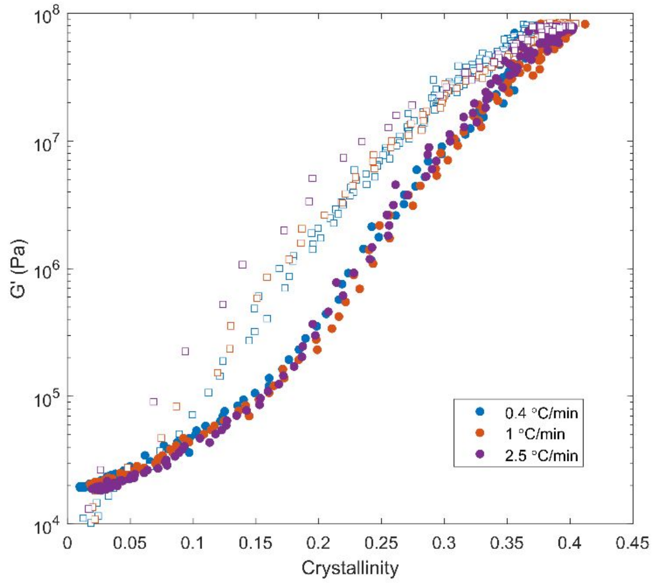

Figure S12. Rate dependent melting hysteresis plot. Three heating rates are shown, (0.4, 1, and 2.5) °C/min. A greater amount of hysteresis is achieved at the fastest heating rate. We attribute this to a lack of thermal equilibration during the temperature ramp. The hysteresis during melting (open symbols) is similar when the ramp rate is 0.4 °C/min and 1 °C/min, which indicates that a temperature ramp rate of 1 °C/min is appropriate to capture hysteresis without significant thermal lag artefacts.

Disclaimer

Certain commercial equipment, instruments, software or materials are identified in this paper in order to adequately specify experimental procedure. Such identification does not imply recommendation or endorsement by the National Institute of Standards and Technology, nor does it imply that the materials or equipment identified are necessarily the best available for the purpose.
Official contribution of the National Institute of Standards and Technology; not subject to copyright in the United States.
